# Efficacy of Sustained-Release Formulation of Moxidectin (Guardian SR) in Preventing Heartworm Infection over 18 Months in Dogs Living in a Hyperendemic Area

**DOI:** 10.3390/ani14203001

**Published:** 2024-10-17

**Authors:** Agustina Isabel Quintana-Mayor, Elena Carretón, José Alberto Montoya-Alonso

**Affiliations:** 1 “El Parque” Veterinary Center, C/ María Encarnación Navarro no. 42 Local Bajo, 35200 Telde, Spain; clinica_el_parque@hotmail.com; 2 Internal Medicine, Veterinary Medicine and Therapeutic Research Group, Faculty of Veterinary Medicine, Research Institute of Biomedical and Health Sciences (IUIBS), Universidad de Las Palmas de Gran Canaria (ULPGC), 35016 Las Palmas de Gran Canaria, Spain; alberto.montoya@ulpgc.es

**Keywords:** *Dirofilaria immitis*, heartworm, chemoprophylaxis, canine, macrocyclic lactones, moxidectin, microspheres

## Abstract

This study investigates the efficacy of a sustained-release (SR) moxidectin microsphere formulation in preventing canine heartworm infection over 18 months in Canary Hound dogs, a hunting breed common in the Canary Islands, which is a hyperendemic region. These dogs typically do not receive preventive treatments and act as reservoirs for the disease. This field study was conducted across 11 hunting kennels with 109 dogs living outdoors, none of whom were receiving heartworm prophylaxis, with *Dirofilaria immitis* prevalence ranging from 11.1% to 57.1% (average 36.7%). Among these, 20 clinically healthy, heartworm-negative dogs were randomly selected to receive a single subcutaneous injection of moxidectin SR (0.17 mg/kg body weight). Antigen and Knott’s tests were performed at 6, 12, 18, and 24 months. All dogs completed the study without adverse reactions and remained heartworm-negative throughout. By the end of the study, kennel heartworm prevalence ranged from 14.3% to 46.7% (average 35.4%). A single subcutaneous dose of moxidectin SR at the recommended dosage may prevent patent heartworm infection in dogs for up to 18 months in hyperendemic regions. Further studies are required to confirm these findings. Extending the efficacy period of moxidectin could improve owner compliance, particularly among those with lower animal health awareness.

## Simple Summary

This study evaluates the efficacy of a sustained-release (SR) moxidectin microsphere formulation in preventing canine heartworm infection over an 18-month period in Canary Hound dogs. Conducted in a hyperendemic region, the study included 109 outdoor dogs from 11 kennels, with heartworm prevalence rates ranging from 11.1% to 57.1%. Twenty healthy, heartworm-negative dogs received a single subcutaneous injection of moxidectin SR. Antigen and Knott’s tests were conducted at 6, 12, 18, and 24 months, and no infections were detected. All treated dogs remained healthy and heartworm-negative throughout the study. The results suggest that a single dose of moxidectin SR may prevent heartworm infection for 18 months; however, further research is needed to confirm these findings. Extending the efficacy interval of moxidectin may enhance owner compliance, especially in populations with lower awareness of animal health.

## 1. Introduction

Heartworm disease (*Dirofilaria immitis*) is a parasitic zoonosis with global distribution, particularly prevalent in temperate, tropical, and subtropical regions due to the climatic requirements of its mosquito vectors [1]. However, areas with warm summers, large rivers, lakes, or extensive irrigation systems also provide ideal mosquito habitats, leading to high prevalence rates [2]. The incidence of heartworm has dramatically increased in recent years, spreading to colder regions, likely influenced by climate change and global warming [3]. This has led to longer mosquito activity periods, accelerated larval development stages, and expanded transmission across multiple regions. Other anthropogenic factors, such as urban heat islands, the introduction of new mosquito species capable of acting as vectors, and increased movement of reservoir dogs, further facilitate this spread [4,5].

The Canary Islands have long been considered a hyperendemic area for heartworm [6,7,8,9,10,11,12]. Veterinarians often prioritize heartworm in the differential diagnosis of cardiopulmonary symptoms in dogs not receiving regular chemoprophylaxis. In Gran Canaria, the prevalence of heartworm has significantly declined since the publication of the first epidemiological studies, from 67.02% in 1994 to 16.03% in the most recent reports, largely due to the continued awareness efforts of veterinarians [6,12]. However, despite this reduction, the prevalence of heartworm has remained stagnant at 16–20.7% for over 15 years. This suggests that further reducing the disease’s incidence on the islands, particularly in Gran Canaria, may be difficult [7,8,9,10,12].

A key reason for the persistently high prevalence of heartworm, despite educational efforts, may be the handling of dogs by hunters and rural populations. These dogs, primarily of the Canary Hound breed, have shown heartworm prevalence rates twice as high as those of the general dog population in Gran Canaria, remaining slightly above 40% in all published studies [8,9,10]. This elevated prevalence is likely due to factors such as the absence of chemoprophylaxis, poor sanitary conditions, outdoor housing on farms or in kennels, and exposure to mosquito bites [10]. Consequently, this breed acts as a reservoir for heartworm, complicating control efforts in the Canary Islands.

Previous studies have assessed the efficacy of sustained-release (SR) moxidectin microsphere formulations, which are used to provide 12 months of protection against heartworm [13,14]. These studies have also indicated that injectable moxidectin results in higher compliance with heartworm prevention [15,16], although the duration of protection remains uncertain, with some suggesting it could last longer [13]. Therefore, this study aims to evaluate the efficacy of a moxidectin SR formulation over 18 months in a hyperendemic area using the highly exposed Canary Hound breed.

## 2. Materials and Methods

### 2.1. Location and Climate of the Canary Islands

Gran Canaria is part of the Canary Islands, a volcanic archipelago located 97 km off the coast of the Sahara. The island features a variety of climates based on altitude and geographical position, including desert (BW), steppe (BS), temperate with hot and dry summers (Csa), and temperate with dry and warm summers (Csb) climates, as per the Köppen climate classification system [17,18]. Gran Canaria is considered a hyperendemic area for heartworm, with prevalence variations depending on the island’s isoclimatic zones [10,11,12].

### 2.2. Studied Animals

**Figure 1:**
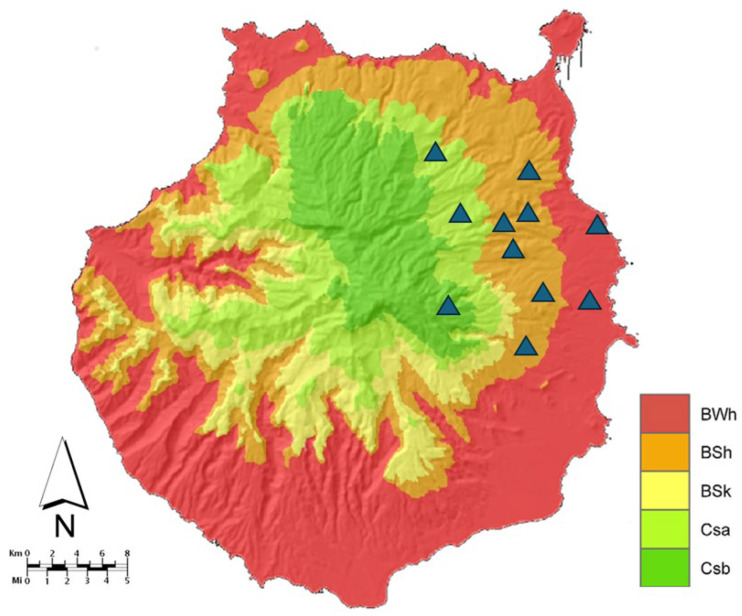
Map of Gran Canaria with the geographical distribution of the sampled kennels. Kennels are marked as blue triangles. Legend: BWh (hot desert climate), BSh (hot steppe), BSk (cold steppe), Csa (temperate with hot and dry summers), Csb (temperate with dry and warm summers). Map of Gran Canaria with the Köppen–Geiger climate classification extracted and modified from the Climate Atlas of the Archipelagos of the Canary Islands, Madeira and the Azores, with permission [18].

This observational field study was conducted across 11 kennels housing Canary Hounds (Figure 1). Inclusion criteria required that the dogs had not received any chemoprophylaxis for heartworm or mosquitoes and lived outdoors continuously.

At the study’s outset, each kennel housed between 6 and 14 dogs, with a total of 109 dogs (Table 1). Blood samples were drawn from the cephalic vein of each dog to determine heartworm prevalence through antigen detection using a commercial test (Uranotest^®^ Dirofilaria, Uranovet, Barcelona, Spain) following the manufacturer’s instructions. Additionally, microfilariae detection was performed using the modified Knott’s test.

Twenty dogs were randomly selected from the 11 participating kennels (1–3 dogs per kennel). The selected animals were clinically healthy and negative for circulating *D. immitis* antigens and microfilariae. Each dog (13 females and 7 males, aged between 7 months and 8 years) received a subcutaneous injection of moxidectin SR (Guardian^®^ SR, Elanco, Spain) at 0.17 mg/kg body weight (0.05 mL/kg body weight). None of the dogs experienced adverse reactions following moxidectin SR administration. All dogs continued their regular activities, including hunting.

Antigen detection and Knott’s tests were repeated at 6, 12, and 18 months (in the 20 treated dogs) and at 24 months (in all dogs). A second dose of moxidectin SR was administered at 24 months in the 20 study dogs. No additional curative or preventive products were given to any of the dogs. All animals were vaccinated annually for rabies. Owners were informed and provided consent for the participation of their dogs in the study.

## 3. Results

At the start of the study, 109 dogs were sampled, revealing an overall heartworm prevalence of 36.7% (40/109). Prevalence varied by kennel, ranging from 11.1% to 57.1% (Table 1).

All 20 dogs finished the study and remained healthy and negative to the antigens and Knott’s tests throughout the study in months 6, 12, 18, and 24. The number of dogs per kennel fluctuated due to deaths (*n* = 13), losses (*n* = 8), sales/leases (*n* = 7), and new acquisitions (*n* = 32). By the end of the study, kennel populations ranged from 7 to 15 dogs, with a total of 113 dogs. The overall prevalence dropped slightly to 35.4% (40/113), varying by kennel from 14.3% to 46.7% (Table 1).

## 4. Discussion

The heartworm prevalence observed in this study aligns with previous findings, indicating that Canary Hound dogs exhibit a higher prevalence compared to the general dog population in Gran Canaria. The prevalences varied in each of the kennels, in some cases increasing and in other cases decreasing, which was probably due to the movement of animals during the study (deaths, losses, sales, leases, and new acquisitions). In a 2016 study, the heartworm prevalence in Canary Hounds was approximately 40%, compared to 20.7% in the general dog population [10]. Similar results were reported in studies conducted between 2000 and 2011, where heartworm prevalence in Canary Hounds ranged from 40.42% (2000) to 43% (2011), while the general dog population showed a decline from 30.19% to 19% during the same period [8,9]. As discussed by previous authors, this is probably due to the fact that these dogs live in unhygienic conditions and do not receive any vaccination, deworming, or chemoprophylaxis against heartworm, in addition to a higher exposure to the vector and the fact that they mainly inhabit the climatic zones with the highest heartworm prevalences, which are the Csa, Csb, and BS climates [10,19].

In these circumstances, Canary Hounds act as a natural reservoir for the disease and act as a barrier to the overall prevalence of the infection on the islands, which remain hyperendemic despite general awareness and the efforts of veterinarians and dog owners. Therefore, it is imperative to seek chemoprophylactic methods that will lead to greater compliance by the owners of these animals in order to help control heartworm in this reservoir animal sector. In this regard, other studies have shown that moxidectin SR offers very satisfactory results in terms of compliance, as observed in the present study, where all dogs completed the study and owners agreed to a second dose of moxidectin SR [15,16]. 

Contrary to what has been observed in other studies, no dogs experienced adverse reactions following administration of moxidectin SR. These results are in line with those published in another large study, which defined the incidence of adverse reactions to a similar moxidectin SR preparation as 14.3/10,000 doses (0.143%) [20]. However, in the study published by Vercelli et al. (2022) [21], it was observed that 12.7% (53/418) of dogs receiving moxidectin SR for the first time experienced adverse reactions (such as mild and temporary discomfort at the injection site, as well as local swelling in the muzzle, paws, eyelids, and lips or general allergic responses such as hives and itching) [21]. The reasons for these differences in the prevalence of adverse reactions are unclear but may be due to the different commercial preparations of moxidectin SR used in the studies. Both studies agreed that adverse reactions were more common in young dogs [20,21]; however, this did not appear to be reflected in the animals in this study.

As adult parasites are not detectable by antigen testing until 5–6 months after infection [1,2,22], testing at 6 months excluded infection prior to moxidectin SR administration as the dogs were not on preventive treatment. Similarly, the subsequent tests excluded infection during this study. Finally, the test performed at 24 months excluded the possibility of infection at least until 18 months after the administration of moxidectin SR. This methodology was similar to that developed by other authors [13], who reported the efficacy of moxidectin SR for the prevention of heartworm in dogs for 12 months.

Moxidectin is a macrocyclic lactone whose potent chemoprophylactic activity against heartworm has been widely demonstrated in different formats [16,23], including against some resistant strains [24,25]. The development of the injectable moxidectin SR formulation provided an interesting alternative to monthly drugs, initially demonstrating that a single subcutaneous injection was effective in protecting dogs against patent heartworm infection for at least 180 days after treatment [26], while subsequent studies have established the efficacy of injectable moxidectin, suggesting efficacy for up to 12 months [13,14]. However, efficacy over a longer period has never been demonstrated. The results of this study showed, for the first time, that the administration of moxidectin SR at the recommended dose could have a chemoprophylactic effect against *D. immitis* infection for 18 months. The possibility of prolonging the efficacy interval of moxidectin SR allows for a higher compliance among pet owners, especially in those groups with less awareness of animal health, which could contribute to reducing the overall heartworm prevalence in dogs in Gran Canaria.

This study is limited by the fact that the dogs studied could not have been bitten by infected mosquitoes during the duration of the study. More specifically, kennels were selected that were located in the most endemic areas of the island, so that dogs living outdoors 24 h a day would have a very high risk of being infected, which is reflected in the high prevalences found in these kennels. Another limitation of the study is that the sensitivity of the antigen detection test used in the study is 94.4% (compared to necropsy), so, although unlikely, false negatives could be possible in some of the dogs studied. The test used in this study has the advantage of detecting antigens that are not associated with the female genital tract of the parasite, i.e., it detects both males and females, although studies have shown that in hyperendemic areas, all infected dogs most likely have female infections [27]. Finally, the limited number of animals studied makes it necessary to extend this research to a larger number of dogs in order to confirm the duration of the chemoprotective effect.

## 5. Conclusions

Based on these results, it can be concluded that a single dose of subcutaneous, injectable moxidectin SR at the package insert-recommended dose (0.17 mg/kg; 0.05 mL/kg body weight) may be effective in preventing patent heartworm infection for at least 18 months in dogs living constantly exposed to mosquito vectors in hyperendemic areas. However, further research in larger numbers of animals is needed to confirm these results. Furthermore, if these results are confirmed, the use of this chemopreventive protocol could contribute to a reduction in heartworm prevalence in hyperendemic areas.

## Figures and Tables

**Table 1:** Details regarding the selected kennels, the climate, the number of dogs for each kennel, and the prevalence of heartworm.

			Beginning of Study			End of Study		
Kennel	Climate	Studied Dogs	*N*	+ *D. immitis*	%–CI (95%)	*N*	+ *D. immitis*	%–CI (95%)
1	BS	3	12	5	41.7–(14.8–70.7)	11	5	45.4–(17.9–70.5)
2	BS	2	9	3	33.3–(12–64.5)	9	4	44.4–(8.7–64.7)
3	BS	3	8	3	37.5–(7.3–73.8)	9	4	44.4–(8.7–64.7)
4	BS	1	9	2	22.2–(0.1–56.9)	9	4	44.4–(8.7–64.7)
5	BW	2	9	2	22.2–(0.1–56.9)	10	3	30–(4.8–66.6)
6	BS	2	10	4	40–(10.5–72.1)	10	3	30–(4.8–66.6)
7	BW	1	6	1	11.1–(0.1–65.9)	7	1	14.3–(0.3–45.5)
8	Csa	2	12	5	41.7–(14.8–70.7)	13	4	30.8–(11.9–57.2)
9	Csa	1	14	8	57.1–(37.2–75.4)	15	7	46.7–(26.3–68.8)
10	Csb	1	9	3	33.3–(4.1–66.8)	10	2	20–(0–55.8)
11	BS	2	11	4	36.4–(10.3–67.2)	10	3	30–(5.2–60.4)
Total		20	109	40	36.7–(27.6–45.8)	113	40	35.4–(26.6–44.2)

Legend: BW: desert climate; BS: steppe climate; Csa: temperate with hot and dry summers climate; Csb: temperate with dry and warm summers climate; (*N*): number of dogs that participated in the study in each kennel; (+ *D. immitis*): number of dogs positive to the antigen test; (%): prevalence of heartworm; (CI 95%): 95% confidence interval.

## Data Availability

All data generated or analyzed during this study are included in this article. The datasets used and/or analyzed during the present study are available from the corresponding author upon reasonable request.
